# Editorialets

**Published:** 1907-06

**Authors:** 


### Editorialets.

---

THE NEW PRESIDENT of the American Medical Association, elected at the recent meeting at Atlantic City, is Dr. Herbert L. Burrell, of Boston. The A. M. A. will meet next June at Chicago.

---

A BARGAIN.—A fourteen years' location, with good improvements of all kinds, on a six-acre lot; a small stock of drugs, plenty of practice, good pay—all will go cheap for cash. For price, write to R. E. GRIGGS, M. D., Ad Hall, Texas.

---

TO SLEEP.

Thou rest o' th' world, sleep, the most peaceful god,
Who driv'st care from the mind, and dost unload
The tired limbs of all their weariness,
And for new toil the body doth refresh.—*Ovid.*

---

TEXAS QUARANTINE CHANGES.—Dr. J. H. Florence, State Quarantine Inspector at Sabine Pass, has been transferred to Galveston, to take charge of that more important station, relieving Dr. J. P.

Tucker, resigned. Dr. Florence's successor at Sabine Pass is Dr. F. C. Ford, of Houston.

---

THE *Medical Era* of St. Louis, Mo., will conform to its usual custom and issue its yearly series of special gastro-intestinal numbers, embracing July and August. The August issue will be given over entirely to the consideration of every phase of typhoid fever. The series will contain about thirty or forty practical papers, and will contain a large amount of valuable information.

---

IN ERROR.—Our esteemed colleague of the *Texas State Journal of Medicine,* May issue, enumerates the acts of medical legislation of the Thirtieth Legislature, and includes that creating an asylum for feeble-minded children. The act was passed, appropriating $25,000, and making the home an annex of the insane asylum at Austin; but His Excellency very properly vetoed it. He held that it should be a separate institution.

---

WE NOTE with pleasure the advances of the Cincinnati Sanatorium, as shown in their thirty-third annual report. This private hospital, for the care and treatment of persons afflicted with nervous and mental disorders, has just completed the south wing of the main building, which is a very desirable addition to the facilities of the institution. During the year there has been a daily average of ninety-three patients, with 37 per cent of recoveries and a mortality rate of only 5.96 per cent.—*Atlanta Journal Record of Medicine.*

---

DEATH OF THE OSTEOPATHIC BILL IN ILLINOIS.—A bill to place an Osteopath on the Illinois State Board of Health failed of passage in the House, May 2d. Another bill introduced was designed to establish a standard for medical and Osteopathic schools, to license Osteopaths without examination, and to confer on them all the rights and privileges which physicians in the State now have. This bill, like many other osteopathic bills introduced in the Illinois General Assembly since 1897, was killed.—*N. Y. Medical Record.*

---

THE NEW MEDICAL EXAMINATION LAW.—Governor Hughes of this State has signed the bill abolishing the three existing State boards of medical examiners and providing for a single board of examiners to be representative of the various schools. The new board is to be composed of nine members. Under this act the Osteopaths for the first time secure recognition in this State. The bill recognizes as "practicing" Osteopaths the three hundred now exercising their calling in this State, but in future all persons desiring to practice Osteopathy must pass an examination before the State Board of Examiners.—*N. Y. Medical Record.*

---

APROPOS OF JUNE.

"In this existence, dry and wet
Will overtake the best of men,
Some little skift o'clouds'll shet
The sun off now and then.
And maybe whilse you're wundern who
You've fool like lent your umbrell to,
And *want* it, out'll pop the sun,
And you'll be glad you hain't got none!"

—*Riley.*

---

DR. C. E. CANTRELL, of Greenville, Texas, was elected President of the State Medical Association of Texas at the Mineral Wells meeting, May 7-9, by practically a unanimous vote of the House of Delegates. This was a graceful and deserved recognition of his zealous, self-sacrificing labors in the cause of medical legislation, and we extend our congratulations to the "Tall Sycamore of Hunt." Dr. H. D. Barnes, of Tulia, was elected Vice-President; Dr. I. C. Chase was re-elected Secretary; Dr. Joe Becton, of Greenville, was elected to the Board of Trustes, vice Cantrell, President, and Dr. M. Smith, of Sulphur Springs, was elected Treasurer, vice Miller, removed to St. Louis. The Smiths got a little mixed in this connection. The newspapers said M. M. Smith, of Austin, and the *Medical Recorder,* of Shreveport, said it was C. A. Smith, of Texarkana.

